# Microgenomic Analysis in Skeletal Muscle: Expression Signatures of Individual Fast and Slow Myofibers

**DOI:** 10.1371/journal.pone.0016807

**Published:** 2011-02-22

**Authors:** Francesco Chemello, Camilla Bean, Pasqua Cancellara, Paolo Laveder, Carlo Reggiani, Gerolamo Lanfranchi

**Affiliations:** 1 Department of Biology and CRIBI Biotechnology Center, University of Padova, Padova, Italy; 2 Department of Anatomy and Physiology, University of Padova, Padova, Italy; Buck Institute for Age Research, United States of America

## Abstract

**Background:**

Skeletal muscle is a complex, versatile tissue composed of a variety of functionally diverse fiber types. Although the biochemical, structural and functional properties of myofibers have been the subject of intense investigation for the last decades, understanding molecular processes regulating fiber type diversity is still complicated by the heterogeneity of cell types present in the whole muscle organ.

**Methodology/Principal Findings:**

We have produced a first catalogue of genes expressed in mouse slow-oxidative (type 1) and fast-glycolytic (type 2B) fibers through transcriptome analysis at the single fiber level (microgenomics). Individual fibers were obtained from murine soleus and EDL muscles and initially classified by myosin heavy chain isoform content. Gene expression profiling on high density DNA oligonucleotide microarrays showed that both qualitative and quantitative improvements were achieved, compared to results with standard muscle homogenate. First, myofiber profiles were virtually free from non-muscle transcriptional activity. Second, thousands of muscle-specific genes were identified, leading to a better definition of gene signatures in the two fiber types as well as the detection of metabolic and signaling pathways that are differentially activated in specific fiber types. Several regulatory proteins showed preferential expression in slow myofibers. Discriminant analysis revealed novel genes that could be useful for fiber type functional classification.

**Conclusions/Significance:**

As gene expression analyses at the single fiber level significantly increased the resolution power, this innovative approach would allow a better understanding of the adaptive transcriptomic transitions occurring in myofibers under physiological and pathological conditions.

## Introduction

Vertebrate skeletal muscles are complex organs composed by a variety of cell types besides the typical long, multinucleated cells called myofibers: fibroblasts in the connective layers, endothelial and smooth muscle cells in the vessel walls, nerves, and Schwann cells around the axons and blood cells flowing through the vessels. Even considering only the contractile components, still skeletal muscle appears as a complex and versatile tissue since myofibers possess a wide range of molecular, metabolic and physiological properties, as well as diverse size [1]. Fibers with glycolytic metabolism, best adapted for rapid activity (FG: fast-glycolytic), and fibers rich in myoglobin and oxidative enzymes, specialized for continuous activity (SO: slow-oxidative), are at the extremes of this range. The expression of distinct myosin heavy chain (MyHC) isoforms defines further groups and provides the basis for the current nomenclature of fiber types [2]. The fiber composition of a muscle is determined in part by genetic factors. However, myofibers are not fixed units but are capable of responding to functional demands by changing the phenotypic profile. This functional plasticity involves metabolic changes and the differential expression of MyHC and other myofibrillar proteins, thus allowing fine tuning of the muscle performance [3], [4].

The actual contribution of single myofibers to the muscle transcriptional phenotype may be overshadowed in gene expression studies with whole muscles, just because of the complex anatomy of skeletal muscle and the heterogeneity of myofibers. The problem is exacerbated in pathological states with infiltrating immune cells or replacement of contractile cells by connective tissue, like in muscular dystrophies or during muscle regeneration [5], [6], [7]. In addition, expression profiles of a heterogeneous population of myofibers produce averaged information even if the pathology affects more dramatically a particular fiber type [8]. Understanding which changes in gene expression actually occur in muscle fibers is of great interest to study muscle plasticity in relation to activity, disuse and aging and may also help future developments for the treatment of muscle diseases [9].

The goal of our work was to demonstrate the feasibility of scaling down the phenotypic analysis of skeletal muscle by applying transcriptome profiling to the single fiber level (microgenomics) [10], [11]. Since a change in gene expression is the most immediate reply of muscle to physiological stimuli, this approach allows a wide phenotypic characterization of fiber types. The selected experimental model were single fibers, isolated by enzymatic dissociation [12], [13], [14] from two murine muscles: the white extensor digitorum longus (EDL, fast-glycolitic) and the red soleus (slow-oxidative). Previously, only quantitative real-time PCR (qPCR) has been applied to analyze the expression of mRNA in single fibers [15]. However, the limit of this approach is that only few individual genes are profiled in each study [16]. We show here that transcriptome profiling of single myofibers results in a much greater discrimination power compared to previous studies with whole muscles [17], [18], as many more differentially expressed (DE) genes were found. Microgenomic analyses identified novel transcriptional markers and revealed pathways of muscle fiber plasticity. The emerging high resolution view of fiber types suggests complex regulation mechanisms particularly in SO myofibers.

## Results and Discussion

### Microgenomics in skeletal muscles

**Figure 1:**
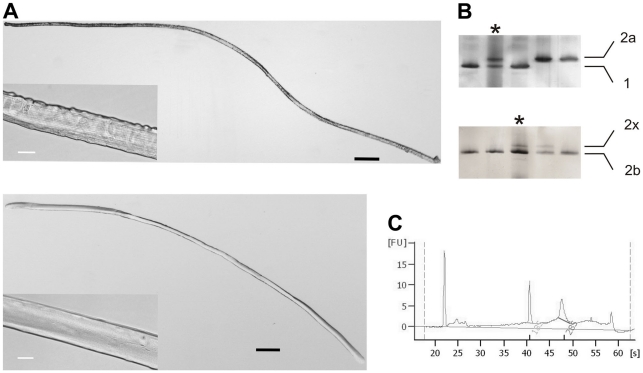
Experimental set up of microgenomic technologies in skeletal muscles. **A**) Transmitted light images at 2.5X magnification of isolated muscle fibers from soleus (top) and EDL (bottom). Intact, unblemished myofibers appears as translucent cylinders. The inset shows details of the characteristic striated pattern (magnification 40X). Black scale bars: 250 µm; white scale bars: 25 µm. **B**) MyHC electrophoretic characterization of single fibers fragments from soleus (top) and EDL (below) muscles. A whole muscle sample has been used as marker of molecular weight (*). As shown in the examples, type 1 and type 2A fibers are abundant in the slow soleus muscle; type 2B and hybrid 2B/2X fibers are most frequent in the fast EDL muscle. **C**) Electropherogram of total RNA extracted from a single soleus myofiber, analyzed in the Agilent 2100 Bioanalyzer using a RNA 6000 Pico LabChip. About 1/3 of the full amount recovered was loaded in this experiment. The high quality of total RNA is confirmed by the presence of ribosomal peaks with no shift to lower fragments (RNA degradation) and no additional signals (DNA contamination).

**Figure 2:**
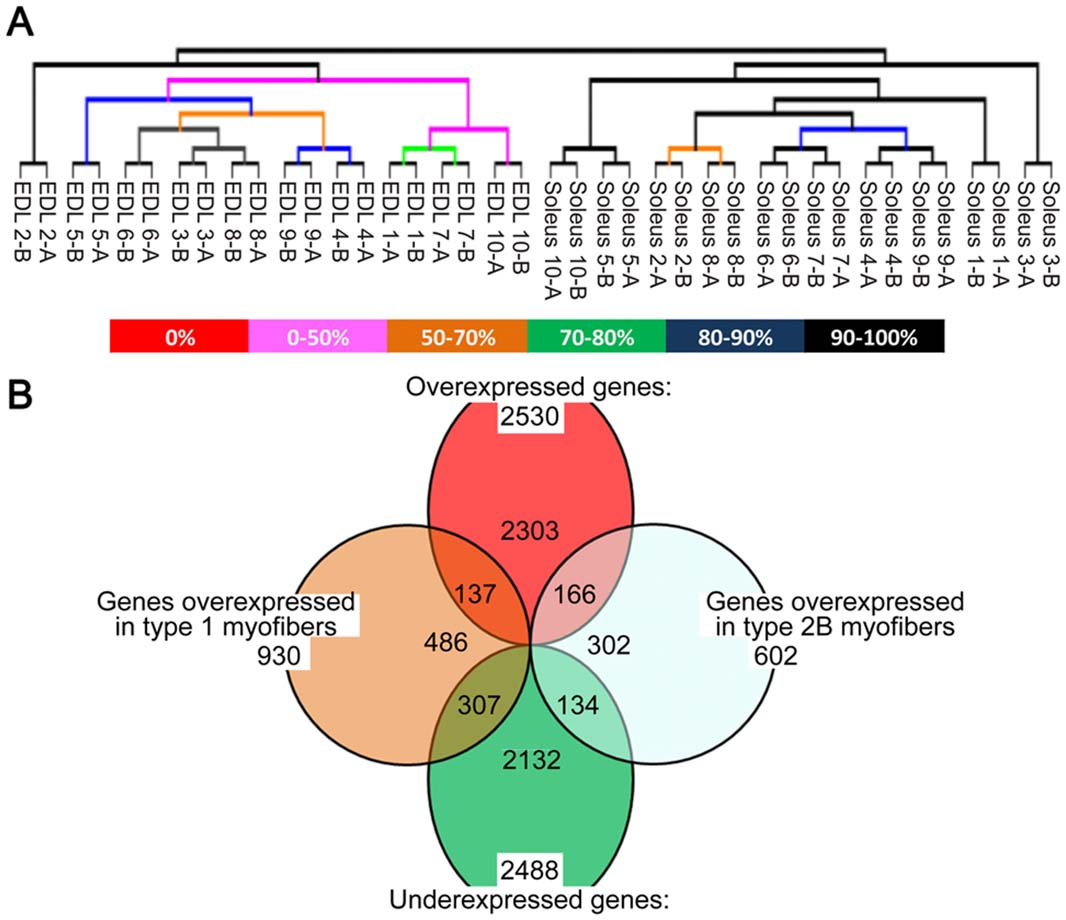
Statistical analysis of microarray data. **A**) Dendogram obtained by hierarchal clustering of expression data generated by 10 pure fibers expressing MyHC-1 (soleus) and 10 pure fibers expressing MyHC-2b (EDL). Microarrays mRNA expression profiling permitted a clear distinction between type 1 and type 2B fibers. Furthermore, technical replicas grouped together within each experiment, confirming the good quality of microarray data. Analysis performed with MeV tool on the set of 11,964 probes that passed the normalization and filtering steps, using Pearson correlation distance. EDL samples came from mice number 1 (1–2), 2 (3–5), 3 (6–8) and 4 (9–10); soleus samples from mice 5 (1–2), 6 (3–5), 7 (6–7) and 8 (8–10). The letters a, b refer to spot replicates present in each microarray slide. **B**) Venn diagram formed by DE genes identified after SAM analyses. Ovals: one-class test; circles, two-class test. Overlapping areas represent genes positive to both tests. FDR values were 0.15% in the one-class test and 0.21% in the two-class test.

Single fibers belonging to two populations of murine muscle fibers, type 1 and type 2B (SO fibers expressing MyHC-1 and FG fibers expressing MyHC-2b, respectively) were selected for gene expression profiling. Although fibers of the two groups could be easily harvested from the soleus and EDL muscles respectively, it was necessary to identify them among the other fiber types. To do this, electrophoretic separation of MyHC isoforms, the gold-standard method for fiber typing, was applied. Briefly, muscles were incubated with collagenase to dissociate intact, unstrained myofibers that were separated under stereo microscope from hyper-contracted fibers (Figure 1A). Isolated myofibers were divided in two parts: one was immersed in Laemmli buffer for fiber typing; the other was placed in RNA extraction buffer. Once identified by SDS-PAGE (Figure 1B), only fibers with the required myosin composition were further processed. The amount of total RNA extracted from a single fiber was obviously very low (Figure 1C) and so two rounds of linear amplification were necessary before mRNA expression profiling. Antisense RNA (aRNA) was amplified from both single fibers and from a reference preparation obtained by mixing together EDL and soleus RNA, thus containing mixed fiber types and non-muscle cells (described in Materials and Methods). Competitive hybridizations were carried out on oligonucleotide arrays; processing of the expression data generated ratio intensities between samples and control, with positive values corresponding to genes more expressed in myofibers. To allow solid statistics of microarray data, we profiled ten type 1 and ten type 2B myofibers. According to the screening procedure (Figure S1), each single individual mouse contributed with 2–3 pure type 1 or type 2B fibers. We assumed that each sample of fibers is as an independent biological replicate and we tested the degree of divergence in gene expression between SO and FG fibers by performing a cluster analysis (Figure 2A). Results suggested that 1) the diversity between type 1 and type 2B fibers could be unambiguously identified at the transcriptional level, since all EDL data formed a distinct group, clearly separated from the group of soleus data; 2) individual donor mice had no effect on formation of subgroups within fiber types, confirming our initial assumption; 3) experiments were of good quality, because technical replicates produced consistent results.

### Removal of non-muscle cells and enrichment for muscle specific genes

**Figure 3:**
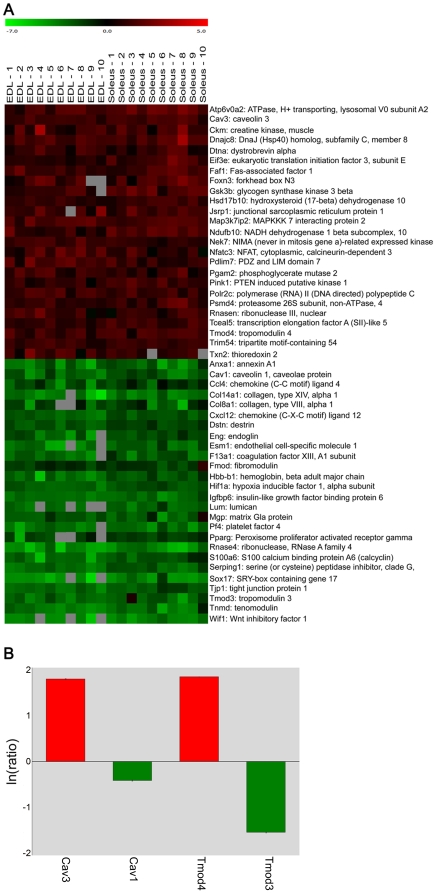
Single fiber analyses allowed removal of non-muscle cells and enrichment for muscle specific genes. **A**) Heat map of selected DE genes identified by one-class SAM analysis. Expression data are Log2 signal ratios values (see Dataset S1) which were converted to colors according to the bar shown at the top: positive values correspond to genes over-expressed in isolated myofibers (red), whereas negative values refer to genes over-expressed in whole muscles (green), and therefore under-expressed in myofibers. Mean values were calculated for two spot replicates. **B**) Validation by qPCR of four DE genes identified by one-class SAM analysis. Signal ratios (natural log values) were calculated independently in pools of 50 type 1 and 50 type 2B myofibers compared to the whole muscle control. The bars in the histogram correspond to the arithmetic mean of the two values separately calculated for type 1 and type 2B fibers. Normalization is relative to two internal references Mfn1 and Txn1; the vertical bars symbolize the intra-assay SD. Positive values correspond to genes over-expressed in myofibers (red bars), and negative values in whole muscles (green bars), as in the heat map.

One-class SAM analysis, carried out on the results of the competitive hybridization of single fibers vs. reference preparation, revealed genes with significantly different expression between myofibers and whole muscle. In total, 2,530 up-regulated and 2,488 down-regulated genes were identified (Figure 2B) using a stringent threshold value to minimize the number of false positives (FDR below 0.25%). Genes highly expressed in non-muscle cells appeared down-regulated in our experimental design and we queried biological databases to gain information about their cellular role. Gene Ontology (GO) enrichment (Table 1) confirmed the presence of entire families of genes coding for proteins expressed in non-muscle cells: globins, immunoglobins, chemokines, interleukins, and coagulation factors of blood cells; collagens, metalloproteases, and proteoglycans occurring in the connective tissue, as well as known markers of endothelial cells (endoglin, endothelial cell-specific adhesion molecule, several gap junction proteins) or Schwann cells (Mog, Plp1). Selected examples are presented in Figure 3A. We noticed some interesting discrepancies between profiling experiments carried out with single fibers and whole muscle organs. For example, a comparison between murine slow and fast muscles showed that the extracellular matrix proteins fibromodulin (Fmod) and matrix Gla protein (Mgp) have a higher expression in the soleus [17]. The same genes were found down-regulated in single fibers (Figure 3A), thus indicating that the difference was not attributable to muscle fibers but to a different contribution in fibroblasts. Importantly, the problem of cellular heterogeneity is possibly emphasized in muscle pathology [6].

**Table 1:** Functional classification of DE genes identified by one-class SAM analysis.

Genes over-expressed in myofibers		
*Category*	*Number of genes*	*P-value*
**Mitochondrion**	**243**	**9.26E-11**
**Cytosol**	**169**	**9.40E-09**
**Contractile fiber part**	**39**	**1.75E-05**
**Sarcoplasmic reticulum**	**17**	**2.00E-04**
**Ribosome**	**76**	**3.00E-04**
**Proteasome complex**	**17**	**7.70E-03**
Other significant	1192	
Not significant	562	
Without ontology	368	

Genes over-expressed in whole muscles		
*Category*	*Number of genes*	*P-value* (*Score*)
**Extracellular region**	**325**	**9.00E-04**
*Sub-categories*		
Extracellular matrix	79	(50.63)
Metalloprotease	45	(3.53)
Inflammatory response	41	(12.10)
Cytokine	40	(9.45)
Cell adhesion	39	(9.69)
Collagen	27	(11.69)
ECM-receptor interaction	20	(6.83)
Innate immune response	16	(3.49)
Blood coagulation	14	(4.33)
Proteoglycan	13	(3.64)
**Endoplasmic reticulum**	**178**	**1.10E-03**
*Sub-categories*		
Cytochrome P450	54	(11.58)
Glycoprotein	53	(1.85)
Golgi apparatus	26	(3.94)
**Membrane**	**1009**	**4.80E-05**
*Sub-categories*		
Cell adhesion	72	(6.88)
Immunoglobulin	56	(5.08)
GPI-anchor	35	(4.15)
Transmission of nerve impulse	35	(2.52)
Not significant	977	
Without ontology	238	

Functional classification of 5,018 DE genes identified by one-class SAM analysis (FDR below 0.25%). GO enrichment was performed with the GOTM tool: general categories were identified, which are shown in bold letters and are associated to P-values (the lower, the better). Several sub-categories were further identified with the DAVID tool, which are associated to a score number (the higher, the better). Additional information in Dataset S2.

A high number of genes up-regulated in myofibers define the identity of muscle cells. GO analysis showed the significant enrichment in genes coding for mitochondrial and cytosolic proteins, as well as for typical muscle structural proteins and muscle specific isoforms of metabolic enzymes (e.g. creatine kinase, enolase, phosphofructokinase) (Table 1). Novel findings were the marked expression of different isoforms in the caveolin, synaptotagmin, and tropomodulin families (Figure 3A), suggesting that muscle cells express specific isoforms also for proteins with a broad range of cellular functions. qPCR confirmed that indeed Cav3 and Tmod4 are up-regulated in myofibers, while Cav1 and Tmod3 have preferential expression in non-muscle cells (Figure 3B). Beside its role in endocytosis, caveolin-3 may help targeting of phosphofructokinase to the plasma membrane [19].

### Molecular signatures of individual FG and SO myofibers

**Figure 4:**
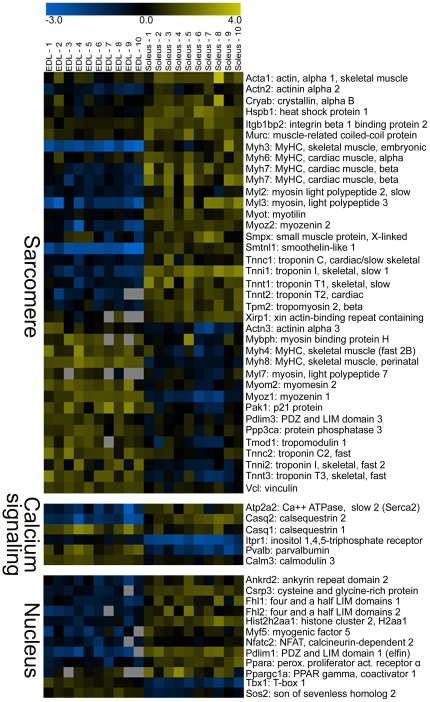
Molecular signatures of fast and slow myofibers revealed by two-class SAM analysis. Expression data are Log2 signal ratios values (see Dataset S1). The different color code emphasizes distinction of fiber types: positive values are in yellow and negative values in blue. Genes with differential expression between type 1 (soleus) and type 2B (EDL) myofibers were grouped according to functional classification: *i*) sarcomeric proteins (GO: contractile fiber part); *ii*) calcium signaling (GO: sarcoplasmic reticulum or calcium binding); iii) nucleus (GO: regulation of transcription or nucleus).

According to our experimental design, all the arrays are independent and all fibers of the same type form a unique class. By running unpaired two-class SAM analysis we focused on gene expression diversity between the two groups of myofibers. In total 1,505 non redundant DE genes were identified in SO type 1 vs. FG type 2B fibers. In particular, 930 probes were over-expressed in type 1 fibers and 602 in type 2B fibers (Figure 2B). Since this number is more than tenfold higher than those previously observed comparing slow and fast muscles [17], [18], it is likely that the single fiber strategy reduces biological noise by subtracting genes expressed in a common set of cell types present in whole muscles [20]. In consequence, the signatures produced with this approach are much richer in muscle-specific and fiber-specific information. A selection of typical muscle genes is presented in Figure 4. We focused our attention to sarcomere and sarcoplasmic reticulum (SR) structures. The higher resolution of microgenomics is evident by looking at the number of distinct components of thick filaments (myosin heavy and light chains) or thin filaments (actin, troponin, tropomyosin) identified with this approach. Of particular interest is the observation that several Z disc proteins were more expressed in type 1 fibers, possibly in agreement with ultra structural studies showing that slow muscles typically show wider Z bands [21]. In muscle cells, the development of the SR requires the increased expression of a medley of different proteins, in part identified by the one-class test (Table 1). Further, electron microscopy has shown that EDL fibers have a more developed SR than soleus fibers [22]. Thus, it is remarkable that only a couple of SR genes were found DE in our study.

To extend the initial analyses to all DE genes, we performed GO enrichment (Table 2). It should be noted that many genes expressed in FG myofibers had no associated description and thus very little information was retrieved for this fiber type. By contrast, several GO functional categories were enriched in SO fibers. A novel and interesting finding was the up-regulation, in SO fibers, of genes coding for proteins involved in the regulation of transcription and RNA processing (Table 2). Among them, we could identify several crucial regulators of fiber phenotype, shown in the heat map of Figure 4. In good agreement with our findings, it is currently believed that calcium-dependent signaling pathways, involving calcineurin, calmodulin-dependent kinases, the transcriptional cofactor peroxisome proliferator-activated receptor-gamma coactivator 1 α (PGC-1 α) and the transcription factor peroxisome proliferator-activated receptor (PPAR) δ, control many of the required changes in gene activity that underlie the conversion to a slow fiber fate [9], [23]. Three closely related subtypes of PPARs regulate the expression of genes involved in respiration and lipid metabolism. PPAR- α plays a major role in fatty acid oxidation and lipoprotein metabolism [24]. Its preferential expression in SO myofibers fits well with our finding that 14 genes of fatty acid metabolism are over-expressed in SO fibers (Table 3). By contrast Pparg (PPAR- γ) was down-regulated in single fibers vs. whole muscle (Figure 3A), as expected for its function in non-muscle cells [25]. We further detected the differential expression of Ppargc1a (PGC-1 α), a master regulator of mitochondrial biogenesis and oxidative metabolism [26]. The up-regulation in type 1 fibers of many mitochondrial proteins (Table 2) and genes of oxidative phosphorylation (Table 3) is in good agreement with this finding.

**Table 2:** Functional classification of DE genes identified by two-class SAM analysis.

Genes over-expressed in type 1 myofibers		
Category	Number of genes	P-value
**Mitochondrion**	**102**	**7.24E-07**
**Contractile fiber part**	**26**	**2.83E-07**
**Ribosome**	**34**	**3.00E-05**
**Other significant**	**464**	
Cytoskeleton	98	(6.13)
Protein complex assembly	30	(3.71)
Ubl conjugation	35	(2.78)
Golgi apparatus	38	(2.50)
Regulation of transcription	85	(2.49)
Chromatin organization	22	(1.96)
Protein transport	45	(1.81)
RNA processing	27	(1.80)
Vesicle	27	(1.71)
Nuclear proteins	51	(1.70)
Not significant	129	
Without ontology	162	

Genes over-expressed in type 2B myofibers		
Category	Number of genes	(Score)
Glycolysis	20	(1.92)
Zinc finger C2H2	25	(1.75)
Proteolysis	44	(1.38)
Other	20	
Not significant	352	
Without ontology	310	

Functional classification of 1,505 DE genes identified by two-class SAM analysis (FDR below 0.25%). GO enrichment was performed with the GOTM tool: general categories were identified, which are shown in bold letters and are associated to P-values (the lower, the better). Several sub-categories were further identified with the DAVID tool, which are associated to a score number (the higher, the better). Additional information in Dataset S3.

**Table 3:** Metabolic and signaling pathways identified at the KEGG bioinformatics resource.

Pathways identified by genes over-expressed in type 1 myofibers		
*Term*	*Count*	*P-value*
**Ribosome**	37	1.42E-08
**Cardiac muscle contraction**	28	1.71E-05
**Oxidative phosphorylation**	37	1.12E-04
**Fatty acid metabolism**	14	1.32E-02

Pathways identified by genes over-expressed in type 2B myofibers		
*Term*	*Count*	*P-value*
**Insulin signaling pathway**	25	8.44E-03
**Wnt signaling pathway**	27	1.36E-02
**Lysosome**	21	3.10E-02
**Glycolysis/Gluconeogenesis**	12	4.58E-02

Pathway analysis of 4,555 significant genes identified by two-class SAM analysis (FDR about 5%). Each pathway is associated to number of genes (count) and P-values (the lower, the better). Additional information in Dataset S4.

A complex network of regulatory proteins governs the expression of muscle genes through combinatorial mechanisms acting on specific DNA elements and in several instances the molecular mechanisms involved in the regulation of fiber phenotype remain unclear [27]. A causal role for muscle regulatory factors (MRFs), key regulators of skeletal myogenesis, in fiber type predisposition has not been demonstrated, although it is known that MyoD is more expressed in fast and myogenin in slow muscles [28]. Here, we found for the first time that Myf5 is up-regulated in SO fibers. A calcium regulated pathway controlling Myf5 gene expression has already been proposed [29]. Ca^2+^ is not only essential for muscle contraction, but it is also a primary signaling molecule implicated in the specification of the slow phenotype [9], [23]. Identification of a calcium dependent regulation of Myf5 expression may further define the mechanism(s) regulating fiber type determination of skeletal muscle. To add further complexity, gene expression programs ongoing in SO myofibers may also recruit nuclear proteins containing PDZ, LIM, or ankyrin domains, and therefore involved in protein-protein interactions. Interestingly, some of them have a dual cellular localization, being also found in the sarcomere (e.g. Ankrd2, Csrp3, Fhl2). The early induction of Ankrd2 and Csrp3 (muscle LIM protein, MLP) genes in response to stretch suggested a role for those proteins in adaptive changes to physical demands [21], [30].

### Pathway analysis of genes expressed in FG and SO fibers

To focus on metabolic differences between fiber types we queried a dedicated resource available at KEGG. Only by lowering the threshold of the statistical test (FDR 5%), thus extending the analysis to 4,555 genes, we could obtain significant results. Almost all genes in the glycolytic pathway that converts glucose into pyruvate were identified as over-expressed in type 2B fibers while many genes of oxidative phosphorylation and fatty acids oxidation were over-expressed in type 1 fibers (Table 3). To our knowledge, this is the first report where fiber specific genes are presented in the context of a genomic network and this is definitely due to the increased resolution achieved moving from comparison between muscles to comparison between individual fibers. Importantly, we could also recognize many components of signaling cascades (Insulin and Wnt signaling pathways) that were expressed more strongly in type 2B fibers.

### Novel potential markers of fiber type

**Figure 5:**
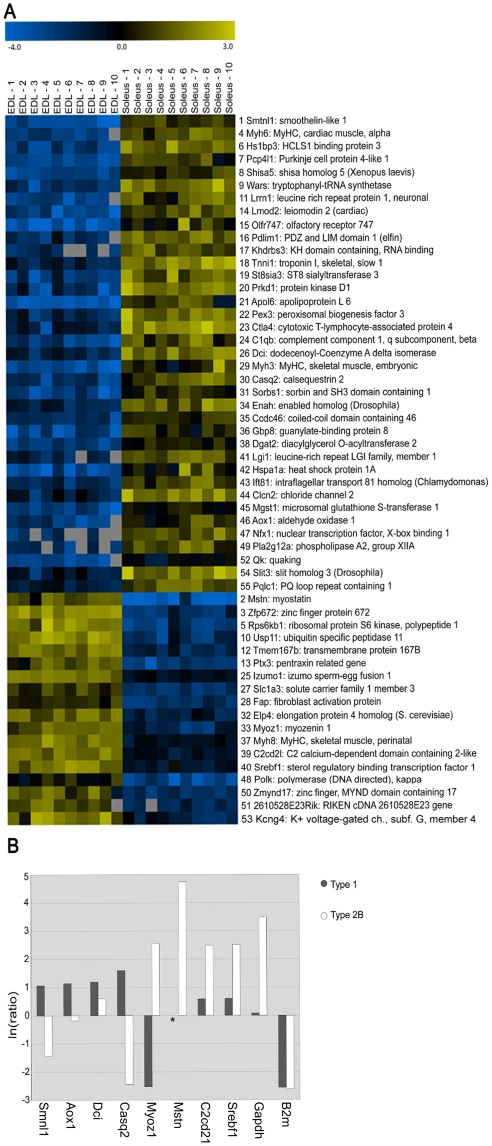
The discriminant analysis emphasized the discovery power of single cell analyses. **A**) Discriminant genes identified by PAM tool. Expression data are Log2 signal ratios values (see Dataset S1). Positive values are in yellow color and negative values in blue color (according to the bar shown at the top). Results sorted by ranking were split in two parts, in order to show genes with preferential expression in type 1 (soleus) or type 2B (EDL) myofibers. **B**) Validation by qPCR of DE genes identified by PAM analysis. Signal ratios (natural log values) were calculated independently in pools of type 1 (gray bars) and type 2B (white bars) myofibers compared to whole muscle control. Normalization is relative to two internal references Mfn1 and Txn1; the vertical bars symbolize the intra-assay SD. Note that the expression of myostatin was not detectable in type 1 myofibers (*).

Prediction Analysis of Microarray (PAM) was implemented in order to find which genes are most useful to discriminate between the two groups of myofibers. The reliability of the PAM test was supported by the presence of well known markers of fiber type. Myostatin, a secreted protein that inhibits muscle differentiation and growth, is strongly associated with MyHC 2b expression in normal muscle [31]. The Myoz1 gene belongs to a family of calcineurin-interacting proteins and several lines of evidences suggest that Myoz1 is expressed exclusively in fast-twitch muscle, while the highly similar protein Myoz2 is found in slow-twitch skeletal muscle and in the heart [32], [33]. Calsequestrin is the most abundant Ca^2+^ binding protein in the SR of skeletal muscle. Two calsequestrin genes encode different isoforms: Casq2 is expressed in slow skeletal and cardiac muscle, while Casq1 is DE between fast and slow skeletal muscles [34]. However, the discriminant analysis emphasized the power for discovery of single fiber analyses, since we identified many other genes that are usually neglected in expression studies based on tissue homogenates (Figure 5A).

To validate the microarray results by an independent method we carried out qPCR experiments on homogeneous pools of 50 fibers. Real-time PCR needs reference genes of invariant expression as internal control. Two canonical references were discarded: Glyceraldehyde-3-phosphate dehydrogenase (Gapdh) had a high expression in FG type 2B fibres and Beta-2-microglobulin (B2m) in the whole muscle control (Figure 5B). Mitofusin 1 (Mfn1) and Thioredoxin 1 (Txn1) instead fulfilled the required criteria in our experimental conditions.

qPCR results indeed confirmed significant differences in the expression level for most tested genes (Figure 5B): Aox1, Casq2, Dci, and Smtnl1 were preferentially expressed in SO fibers; C2cd2l, Mstn, Myoz1, and Srebf1 in FG fibers. While smoothelin-like 1 (Smtnl1) seems a typical slow gene here, immunohistochemical analysis showed that the corresponding protein is more abundant in fast-oxidative fibers, belonging to the type 2A subgroup [35].

### Concluding comments

The structural variability of muscle fibers has been related to differences in relative proportions of membrane structures and to different expression of regulatory and contractile proteins. Although a number of methods have been applied to investigate muscle fiber heterogeneity (reviewed in [36], the list of genes involved in the molecular and cellular processes associated to muscle properties still need to be clarified and completed. The emerging microgenomic technologies provide fundamental improvements in experimental design, reflecting the real complexity of heterogeneous tissues [20], [37]. In skeletal muscle, the multinucleate myofibers are easily distinguished from the other cell types and we did profit by the large cell size to classify them according to the expressed MyHC isoform. Subsequently, fiber-specific genes were linked to MyHCs by rules of co-expression. Our results showed that a great number of genes are indeed DE between fibers within a muscle. As anticipated, comparison between individual fibers greatly increased the resolution of the analysis, with respect to results obtained with mixed fiber populations [38]. We have thus generated the first wide catalogue of gene expression in type 1 and type 2B fibers that is a useful starting point to test novel markers of fiber types and to direct functional studies on the role of poorly characterized genes in the adaptive potential of muscle fibers. In future, it will be interesting to profile fiber types with intermediate characteristics (e.g. hybrid or type 2A and 2D/X fibers).

Importantly, single fiber profiles were virtually free from non-muscle transcriptional activity that was detected in standard muscle homogenates. Primary myogenic cultures are another common model to study muscle physiology and pathology. The problem of cellular heterogeneity might affect also this system, as not all myoblasts differentiate into myotubes and fibroblasts still are a significant fraction of the total cells. A recent survey highlighted differences in gene expression between human muscle biopsies and cultured muscle cells [39]. Enriched categories in myotubes were predominantly related to cytoplasm, endoplasmic reticulum, and extracellular matrix. We showed here that several extracellular matrix genes identified in adult muscle samples are actually expressed in fibroblasts and the same could be true for the in vitro cultured cells. Furthermore, in vitro differentiation of primary myoblasts fails to convert myotubes to mature muscle fibers. Due to inappropriate stimuli (i.e. lack of innervation), cultured muscle cells display reductive metabolic adaptations and activation of atrophy-like processes [39]. By contrast, dissociated myofibers provide a more relevant and accurate culture model for the study of mature skeletal muscle, as showed in the mouse flexor digitorum brevis muscle [40]. The microgenomic technologies further expand the potential of this approach and should make possible to profile almost every muscle fiber type.

The diversity of fiber types is likely regulated by multiple signaling pathways and transcription factors rather than the result of a single ‘master’ switch [27]. Through gene annotation enrichment analysis it is possible to define groups of genes that may share a common regulatory pattern [41]. From the results obtained in our study we hypothesize the following functional units in SO fibers: i) genes of fatty acid metabolism regulated by PPAR-α; ii) slow isoforms of contractile proteins controlled by NFATs; iii) genes of oxidative metabolism promoted by PGC-1α. Genetic programs in FG fibers are at the moment more elusive. Fast glycolytic fibers seem more difficult to examine with this method for technical limitations of different nature. First, more than half of DE genes had no associated GO description (Table 2). Although the ontology vocabulary has been recently enriched with new terms to describe specific muscle structures and biological processes [42], many gene products are still waiting for annotation. A better functional annotation exists for genes implicated in heart disease [43] that in many instances are also expressed in slow skeletal muscles. Second, studies in the rat have shown that type 2B fibers have a lower total RNA content compared to type 1 fibers [44]. Reduced quantities of input RNA may lead to stochastic effects during global mRNA amplification [45], thus lowering the number of DE genes identified by statistic tests. It also possible, however, that gene expression is intrinsically more stochastic in the FG fibers than in other fiber types.

**Figure 6:**
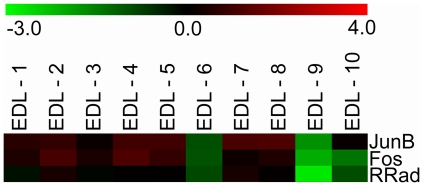
Differential expression among individual fast fibers. Expression levels among individual type 2B fibers of three selected genes (JunB, Fos, RRad). Expression data are Log2 signal ratios values which were converted to colors according to the bar shown at the top: positive values correspond to genes over-expressed in isolated myofibers (red), whereas negative values refer to genes over-expressed in whole muscles (green), and therefore under-expressed in myofibers.

A central issue in single cell biology is that assays of individual cells are expected to produce a high degree of expression repertoires, even in a context of relatively homogeneous cell population [37]. Within our study we indeed found some genes that are expressed in a different fashion between fibers expressing the same MyHC isoform. Noticeably, the expression of the transcription factors JunB, Fos, and RRad (Ras-Related Associated with Diabetes), that are correlated within the insulin pathway in muscle [46], was clearly down regulated only in a small group of type 2B fibers (Figure 6). These results confirm the high resolution power of expression profiles and suggest that genomic data may lead to novel classification systems at the transcriptional level, by discovering subpopulations of genes whose expressions are altered to modify and maintain specific myofiber phenotypes.

Myofibers can adapt their metabolic and contractile properties by switching on and off structural genes, with or without a change in MyHC isoform content [47]. These changes are anticipated at the transcriptional level through the expression of specific transcription factors, chromatin modifiers, cofactor proteins and even miRNAs [48]. Expression of these genes is, in turn, under the control of a complex array of signals that ultimately integrate humoral factors and mechanical stimuli. Our innovative approach is well suited for studies on muscle plasticity, since it produces fiber-specific information and allows for the detection of key components of metabolic and signaling pathways. Also, muscle disorders with marked fiber type specificity have been reported. For example, oculopharyngeal muscular dystrophy is associated with severe atrophy restricted to FG fibers, while oxidative fibers in the soleus muscle are spared [49]. Consistent with fiber type dependent muscle wasting, the ubiquitin ligase MuRF1 was recently shown to be preferentially expressed in FG fibers [50]. Thus, gene expression profiling of single fibers may help studying in deeper detail muscle diseases and pathological states.

In conclusion, the shift from comparison between muscles to comparison between individual fibers has made possible an increased resolution analysis of muscle specific genes. While the knowledge of muscle cells may already benefit of the present study, it is likely that the microgenomic approach will become more and more attractive for studies on muscle heterogeneity, plasticity and diseases, as single cell technologies are rapidly evolving and novel protocols are under development for faster and more efficient analyses [51], [52].

## Materials and Methods

### Ethics statement

All aspects of animal care and experimentation were performed in accordance with the Guide for the Care and Use of Laboratory Animals published by the National Institutes of Health (NIH Publication No. 85-23, Revised 1996) and Italian regulations (DL 116/92) concerning the care and use of laboratory animals. Experimental procedures were approved by the local Ethical Committee of the University of Padova.

### Animals

Wild-type CD1 mice (Charles River) were housed in a normal environment provided with food and water. Adult males were killed by rapid cervical dislocation, to minimize suffering, at three months age (weight: 33–35 g).

### Enzymatic dissociation of myofibers

Detailed information is available about fiber composition and length in the mouse soleus and EDL muscles [8], [53], [54]; a single myofiber is supposed to have about a hundred of nuclei [55]. We modified published methods for long fibers isolation [12], [13], [14], in order to keep the digestion time as short as possible and avoid activation of stress response genes. Muscles from both hind limbs of the same mouse were immediately removed by microdissection, taking care to handle them only by their tendons to minimize mechanical damage to the fibers (see also Figure S1). Digestion proceeded for 40–45 min. at 37°C in 1 ml high-glucose Dulbecco's modified Eagle medium (DMEM; Invitrogen-Gibco) containing 10 mg type I collagenase (220 U mg^−1^; Sigma). The collagenase-treated muscles were sequentially rinsed for 2 min. in 3 ml of DMEM, 3 ml DMEM supplemented with 10% fetal bovine serum (FBS) and 3 ml of DMEM and finally transferred into 50 mm×18 mm well containing 3 ml of DMEM with 10% FBS. All plastic was pre-rinsed with 10% FBS, to prevent sticking. Single myofibers were liberated by gentle physical trituration with a wide-mouth plastic Pasteur pipette (about 4 mm diameter). The triturating process was repeated several times until about 100 intact fibers were obtained. After each physical trituration, the muscles were transferred in a new well, to get rid of collagen wisps and hyper-contracted fibers. Quickly, intact and well isolated fibers were picked under stereo-microscope and washed first in DMEM and then in phosphate buffered saline (PBS; 137 mM NaCl, 2.7 mM KCl, 10 mM Na_2_HPO_4_, 1.76 mM KH_2_PO_4_, pH 7.4). About one-third of each fiber was clipped and placed in Laemmli buffer (for fiber typing by SDS-PAGE, described below); the remaining part of the fiber was dissolved in lysis solution for RNA extraction. All samples were collected within 45 min. from the last trituration step.

### MyHC isoform identification by SDS-PAGE

MyHC isoforms were separated in SDS-PAGE as described by Talmadge & Roy [56]. About one-third of each fiber was solubilized at 90°C for 5 min in 10 µl of Laemmli buffer (Tris pH 6.8 62.5 mM, glycerol 10%, SDS 2%, β-mercaptoethanol 5%). After denaturation in SDS and heat, proteins were analyzed on 4% stacking (4% polyacrylamide 50∶1, 30% glycerol, 70 mM Tris (pH 6.7), 4 mM EDTA and 0.4% SDS) and 8% resolving gels (8% polyacrylamide 50∶1, 30% glycerol, 0.4% SDS, 0.2 M Tris, and 0.1 M glycine). Slabs were 18 cm wide and 16 cm high. Electrophoresis was carried out at 4°C for 43 h, at 100 V for the first 3 h and at 230 constant V for the remaining time. After silver staining (Bio-Rad Silver stain), bands of MyHC isoforms appeared separated in the 200 kDa region and were identified according to their migration rates compared to molecular weight standards. All gels were scanned, digitally stored and analyzed.

### RNA samples preparation

#### RNA extraction

Reagents optimized for minute amount of material assured higher RNA yields compared to the classical Trizol reagent. Total RNA was extracted from fiber fragments or pools using the silica membrane technology of RNeasy Micro Kit (Qiagen). Single fibers were disrupted by adding 75 µl Buffer RLT and lysate was homogenized by vortexing for 5 min. The protocol was essentially that suggested by the manufacturer, with the following modification: RNA elution was performed with 14 µl RNase-free water pre-heated at 37°C and repeated a second time to avoid loss of RNA in the column. Due to the dead volume of the column, we recovered about 20–24 µl. We estimated that the amount of total RNA purified from a single fiber is in the range of one to few nanograms.

#### RNA amplification and labeling

Purified RNA samples were lyophilized and amplified twice using the Amino Allyl MessageAmp™ II aRNA Amplification Kit (Ambion), in accordance with the manufacturer's instructions. First strand synthesis with an engineered reverse transcriptase should produce virtually full-length cDNA, which is the best way to ensure reproducible microarray results. The use of a modified oligo(dT) primer bearing a T7 promoter [57] allows the next amplification steps: after second strand synthesis and clean-up the cDNA becomes a template for in vitro transcription with T7 RNA polymerase. By subjecting the aRNA to a second round of amplification we obtained on average about 80 µg aRNA from type 1 fibers and 45 µg from type 2B fibers. That material was enough to carry out several array hybridizations. 1 µl aRNA sample was quantified using the Nanodrop ND-1000 Spectrophotometer (Celbio) and the same amount was checked for RNA integrity (see below). About 5 µg aminoallyl-labeled aRNA were coupled with Cy5 or Cy3 dyes (GE Healthcare) and purified on column (Ambion).

#### RNA quality control

Both RNA extracted from single fibers (1/3 of total) and aRNA (200 ng) were analyzed using the RNA 6000 Pico or Nano LabChip on a 2100 Bioanalyzer (Aligent). The sample (1 µl) was separated electrophoretically as described by the manufacturer and data were displayed as a gel-like image and/or an electropherogram. All poor quality RNA samples were discarded.

### Experimental design

For competitive hybridizations between single fibers and whole muscles, it was essential to find a control RNA with a balanced composition of type 1 and type 2B fibers. In the mouse, soleus muscles contain 36% type 1 and 59% 2A fibers [54], while EDL are composed by 81% type 2B and 16% 2X fibers [8]. An artificial control was created as follows: three couples of soleus and EDL muscles were removed from 3 different mice and treated with type I collagenase as described above. Total RNA was extracted separately from EDL and soleus muscles using the Trizol protocol (Invitrogen). By mixing about 1/3 RNA from EDL and 2/3 RNA from soleus muscles we balanced the contribution of type 1 and type 2B fibers. The control RNA was amplified and labeled as described above. Unfortunately, competitive hybridizations were afflicted by biased ratio values, due to saturation of high-intensity spots [58]. For about two hundred highly expressed genes, the recorded pixel intensity was truncated when it reached the maximum value in one or both channels. Significant examples included fast SR Ca^2+^ ATPase (Atp2a1, alias Serca1) or Glyceraldehyde-3-phosphate dehydrogenase (see Dataset S2).

### Microarray features

The Mouse Genome Oligo Set (version 1.1, Operon) consisted of 13,443 70mer oligonucleotide probes and it was purchased from the Gene Expression Service available at CRIBI. Each oligo was spotted in two replicates on MICROMAX SuperChip I glass slides (Perkin-Elmer) using Biorobotics Microgrid II (Apogent Discoveries). We produced an updated and careful annotation of all sequences by querying three databases: ENSEMBL (version 56), RefSeq (version 38) and UniGene (version 183). About 1,500 probes did not find significant hits. The updated platform (version 2.0) has been submitted to the GEO Database, with Accession Number GPL10688.

### Microarray experiments

All microarray data is MIAME compliant and the raw data is available in the GEO database (accession number GSE23244).

Labeled targets from single fibers and muscle control were mixed and ethanol precipitated. After dissolving the pellet in 120 µl of hybridization buffer (5X SSC, 0.1% SDS, 25% formamide), samples were denatured at 90°C for 2 min and added to the microarrays. Prehybridization was for 20 hours at 46°C in the presence of 5X SSC, 5X Denhardt, 0.1% SDS, 100 ng/µl ss-DNA. Competitive hybridizations were carried on for 44 hours at 46°C in an ArrayBooster microarray incubator (Advalytix), followed by a series of post-hybridization washings.

### Analysis of microarray data

#### Scanning

Microarray slides were inserted into a VersArray ChipReader dual confocal laser scanner (Bio-Rad) for fluorescence detection at 5 µm resolution.

#### Quantification

Raw scanner images were processed with ScanArray Express Software (Perkin-Elmer) for fluorescent quantification.

#### Normalization

Global mean normalization was performed across element signal intensity and expression values were transformed into Log2 ratio of normalized intensities. Positive values correspond to genes over-expressed in myofibers, whereas negative values refer to genes over-expressed in whole muscles, and therefore under-expressed in myofibers. All statistical analyses were performed with MIDAW [59]. Before proceeding with the SAM tests described below, data were filtered by removing 1,475 probes that were associated to NA spots in more than 60% of experiments.

#### Cluster analysis

Hierarchical cluster analysis was performed by MultiExperiment Viewer (MeV, v4.5.1), a part of TM4 Microarray Software Suite [60]. Support tree was obtained using Pearson Correlation with bootstrapping resampling method. Technical replicas were present in each slide (see Microarray features) and they were split in two subarrays to check the quality of microarray data, as explained in Supporting information (Text S1).

#### Differentially expressed genes

Significance Analysis of Microarrays (SAM) is a non-parametric statistical test based on a permutation approach specifically implemented for microarray data [61]. In one class SAM analysis, all myofibers were assigned to a unique class, thus distinguishing two populations of muscle and non muscle cells. In the two class SAM analysis type 1 myofibers formed one group and type 2B a second group, to find DE genes between the two fiber types. The threshold level is associated to a False Discovery Rate (FDR) value: the lower FDR, the less false positives are expected. FDR values between 1–5% are commonly recognized as highly significant.

#### Functional annotation

Gene Ontology enrichment was performed with the Gene Ontology Tree Machine tool (GOTM) using a P-value of 0.1 [62]. Sub-categories were identified using the Functional Annotation Clustering of the Database for Annotation, Visualization and Integrated Discovery (DAVID v6.7). Gene enrichment in pathways was performed at the DAVID web server [41] using a P-value of 0.5, interrogating KEGG database. In all the analyses platform transcripts were used as background.

#### Analysis of discriminant genes

Supervised class-prediction analyses were performed by applying Prediction Analysis of Microarrays (PAM). This program uses the method of the nearest shrunken centroids to identify a subgroup of genes that best characterizes a predefined class [63].

### qPCR

The details of real-time quantitative PCR (qPCR) were described in a previous study [8]. Experiments were performed in a 7500 Real-Time PCR System (Applied Biosystems), using the SYBR Green chemistry (Finnzymes). RNA was extracted from groups of 10 fibers classified by SDS-PAGE as belonging to the same type, by adding 350 µl Buffer RLT and proceeding as indicated above. The RNA pool contained finally RNA from 50 individual fibers. About one microgram of aRNA was reverse transcribed using Superscript III reverse transcriptase (Invitrogen) according to the manufacturer's directions. Gene-specific primers were selected with Primer 3 software and the specificity of each primer set was monitored by dissociation curve analysis. Samples from pooled fibers and whole muscles (same RNA control of microarray experiments) were amplified from multiple serial dilutions of the cDNA input. Differences in gene expression were evaluated by a relative quantification method [64]. Values were normalized to the mean expression of two different internal reference genes (Mitofusin 1 and Thioredoxin 1), with invariant abundance in our experimental conditions. Normalized ratios were converted in logarithmic scale and standard deviation was calculated according to Marino et al. [65].

## Supporting Information

Figure S1 Flow chart of the experimental strategy.
(PDF)

Text S1 Protocol for microarray sample processing.
(PDF)

Dataset S1 Expression data presented in the main paper.
(XLS)

Dataset S2 Additional information for data presented in Table 1.
(XLS)

Dataset S3 **A**dditional information for data presented in Table 2.
(XLS)

Dataset S4 **A**dditional information for data presented in Table 3.
(XLS)
