# Supplementary material for: Microgenomic Analysis in Skeletal Muscle: Expression Signatures of Individual Fast and Slow Myofibers
Source: PLoS One. 2011 Feb 22;6(2):e16807. doi: 10.1371/journal.pone.0016807 (PMC3043066; doi:10.1371/journal.pone.0016807)
Supplement: Figure S1 — Flow chart of the experimental strategy. (PDF) [file pone.0016807.s001.pdf]

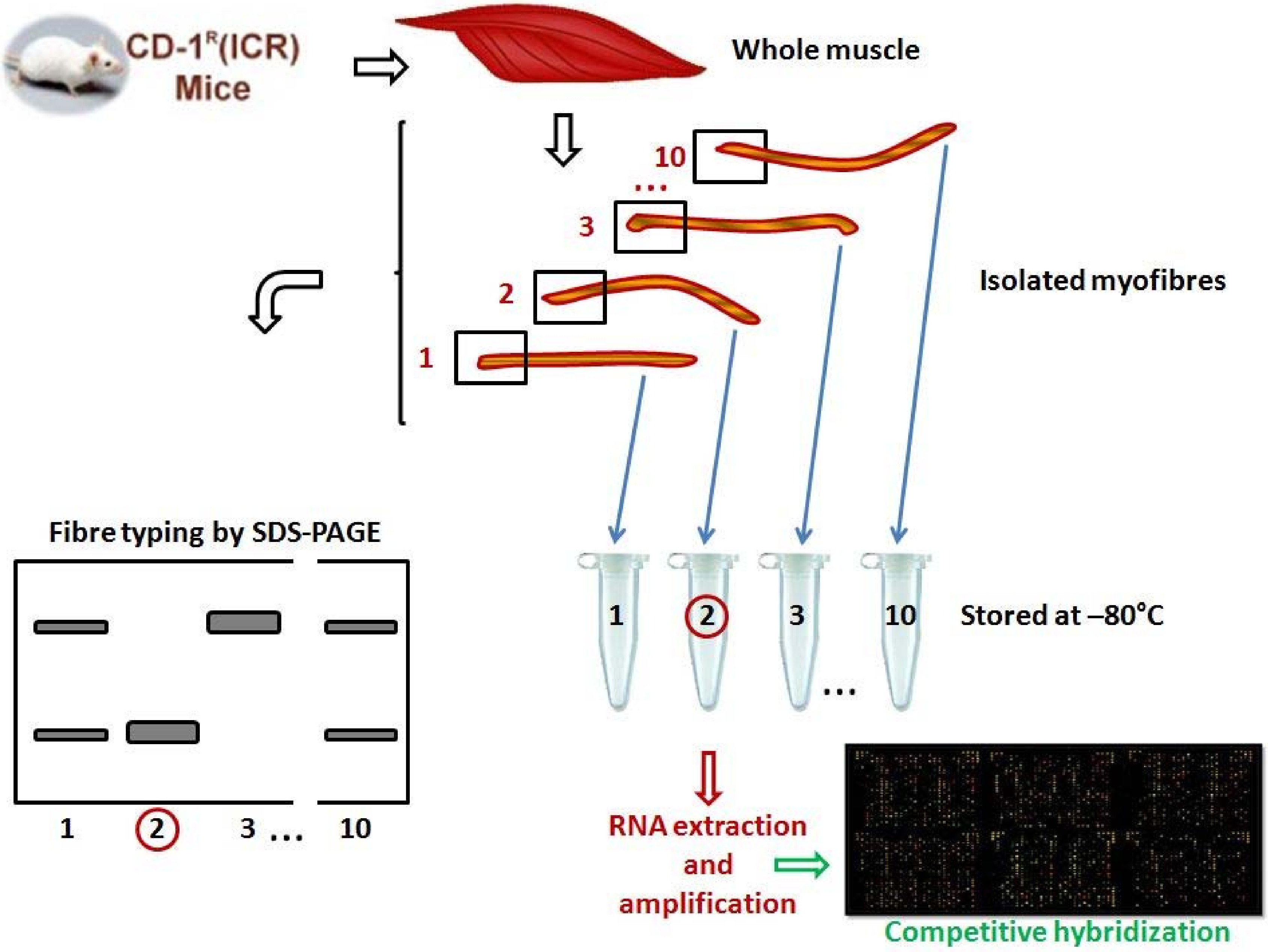
CD-1R(ICR)
Mice
Whole muscle
10
...
3
2
1
Isolated myofibres
Fibre typing by SDS-PAGE
1
2
3 ...
10
1
2
3
10
...
Stored at –80°C
RNA extraction
and
amplification
Competitive hybridization

Figure S1: Experimental design for mRNA expression profiling of type 1 and type 2B myofibers.

One CD1 mouse was sacrificed in each experiment. Either EDL or soleus muscles from both hind limbs were removed and treated with type I collagenase in order to obtain about hundred viable, intact fibers (Methods). Ten well isolated, single fibers were picked under stereo-microscope as quick as possible. Each fiber was cut in two pieces: the smallest part was used for fiber typing by SDS-PAGE (left), while the remaining part of the fiber was stored at -80°C in lysis solution (right). Once type 1 and type 2B fibers were identified according to MyHC protein content, they were further processed for RNA extraction. Finally, mRNA was subject to two rounds of amplification before competitive hybridizations.

For the microarray experiments we collected 10 type 1 and 10 type 2B fibers, after screening 40 soleus and 40 EDL fibers isolated from 8 animals. A single mouse individual contributed with 2 – 3 pure type 1 or type 2B fibers. The same screening strategy was applied to create homogeneous groups of fibers for the next qPCR assays.
