# Supplementary material for: Microgenomic Analysis in Skeletal Muscle: Expression Signatures of Individual Fast and Slow Myofibers
Source: PLoS One. 2011 Feb 22;6(2):e16807. doi: 10.1371/journal.pone.0016807 (PMC3043066; doi:10.1371/journal.pone.0016807)
Supplement: Text S1 — Protocol for microarray sample processing. (PDF) [file pone.0016807.s002.pdf]

Chemello F. *et al* – **Protocol for microarray sample processing**

1. Download the expression data from GEO database (series: GSE23244) and save each single sample as text file. Delete the first 57 rows (comments, starting with the symbol #).

2. Import the data in a spreadsheet and keep the following columns: 1) ID_REF; 2) VALUE; 3) Ch1 % > B + 1 SD; 4) Ch1 % > B + 1 SD.
   #ID_REF = Unambiguous spot ID in our microarray platform
   #VALUE = Log(2) ratio of normalized intensities (data used in our work)
   #Ch1 % > B + 1 SD = the percentage of feature pixels with intensities more than one standard deviation above the background pixel intensity, at wavelength #1 (633 nm)
   #Ch2 % > B + 1 SD = the percentage of feature pixels with intensities more than one standard deviation above the background pixel intensity, at wavelength #2 (543 nm)

3. We applied the following filter: all spots with value lower than or equal to 25 in the Ch1 % > B + 1 SD or Ch2 % > B + 1 SD columns were estimated as not significant. The corresponding numbers in the VALUE column were replaced with NA for the next analyses.

There are two spots of the same probe in our microarray. To split the two technical replicas within each experiment it is necessary to add the "Oligo probe description" from the microarray platform.

- Download the data about our microarray platform from GEO database (accession: GPL10688) and save it as text file. Delete the first 12 rows (comments, starting with the symbol #).

- Import the data in a spreadsheet and keep the following columns: 1) ID; 2) Description.
  #ID = Unambiguous spot ID
  #Description = Oligo probe description

4. Be sure that both data are sorted by ID and paste the "Description" column into the sample file. Sort the data by Description (alphabetical order); at this point we deleted the last rows, containing spike-in controls, empty spots and grid positional probes. Create the two datasets with your favorite method. The final file should have two duplicate values for each of the 13439 probes.

5. Repeat steps 2 – 4 for each sample file.

Data from all sample files were imported in a unique spreadsheet. Before proceeding with the SAM tests, we filtered the expression data by removing probes that were associated to NA spots in more than 60% of experiments.
